# Experiences of antibiotic use among Brazilian healthcare users: An exploratory study

**DOI:** 10.1111/hex.13664

**Published:** 2022-11-24

**Authors:** Luiz F. Zago, Juliana S. Correa, Roberto R. da Silva‐Brandão, Lislaine A. Fracolli, Maria Clara Padoveze, Sandi Michele de Oliveira, Gloria C. Corboda Currea

**Affiliations:** ^1^ Department of Nursing in Collective Health, School of Nursing University of São Paulo São Paulo Brazil; ^2^ Section of General Practice, Institute of Public Health, Faculty of Health and Medical Sciences University of Copenhagen Copenhagen Denmark; ^3^ Antimicrobial Research Unit, School of Health Sciences University of Kwazulu‐Natal Durban South Africa

**Keywords:** antibiotic use, antimicrobial resistance, exploratory research, patient experience, primary care

## Abstract

**Introduction:**

This article analyzes experiences of antibiotic use and bacterial infections among Primary Health Care users of the Brazilian Unified Health System (SUS) and the possible implications for antimicrobial resistance (AMR). The aim is to map aspects that shape users' lay knowledge regarding antibiotics use and AMR.

**Methods:**

This is an exploratory study, which consists primarily of individual in‐depth interviews with 19 respondents. Recurrent interview topics were coded and analysed according to thematic content analysis.

**Results:**

Our findings show users' lived experiences constitute three dimensions related to users' previous antibiotic use: (1) lay knowledge about medicines; (2) previous bacterial infections and (3) communication during the consultation. Lay knowledge encompasses the users' understanding of how antibiotics work in comparison to other drugs and experimentations they make with medication. Users' narratives about bacterial infections are divided into situations of urinary tract infections and antibiotic treatments for other conditions. Communication during the consultation is mainly characterized by a lack of shared knowledge and trust in the doctor–patient relationship.

**Discussion:**

Users bring together knowledge learned from their own experiences to create the rationale, which shapes how they understand antibiotic use, bacterial infections and medical advice. These experiences are interwoven with information received from healthcare professionals (HPs) on these topics, creating a scenario that goes beyond professional information about antibiotic use. Users have knowledge about medication, antibiotics use and bacterial infection but do not have room to share it with HP, allowing lived experiences to take precedence over professional information.

**Conclusion:**

Users ascribe symbolic meanings to antibiotics creating a lay knowledge frame, even if this knowledge is not scientifically correct. The personal experiences of bacterial infections and their treatment are also an important source of knowledge about antibiotic use and AMR among users. Users demand from their HPs both trust and willingness to listen to their health narratives and experiences. By considering lay knowledge as part of the assessment of a user's health condition, rather than dismissing it as erroneous and therefore unworthy of attention, HPs may enhance the compliance of users.

**Patient or Public Contribution:**

Patients or community members did not participate in the design stage of the study. Primary Care patients were invited to participate as respondents of in‐depth interviews, which were carried out by the first author at a Primary Care Unit (PCU) in the suburb of Campo Limpo, Southern region of São Paulo, Brazil. Patients were interviewed after reading and signing a Free and Informed Consent Form, holding with them a copy of the Form. Among the final activities of the project, a feedback session at the same PCU is planned to report on the results of the study. All respondents will have the opportunity to contribute further information regarding their antibiotic use and exchange knowledge and experiences on antimicrobial resistance.

## 1 INTRODUCTION

Antibiotic (mis)use is one of the contributing factors to the increase in antimicrobial resistance (AMR) worldwide, a global health threat.^1^, ^2^ A major approach to defining context‐specific actions to curb the inappropriate use of antibiotics involves knowledge, attitudes and practices (KAP) surveys.^3^, ^4^, ^5^, ^6^ Biomedical information is a criterion to assess which KAP are ‘appropriate’, reinforcing the prevalent notion that healthcare users have ‘knowledge gaps’ regarding antibiotic use and AMR.^7^ The way KAP research is generally developed in analysing AMR and antibiotic use may underestimate the narratives from which the individual's practices and behaviours come and gain meaning.

Taking an approach different from the traditional KAP perspective, this article analyzes experiences of antibiotic use and bacterial infections among Primary Health Care users (hereinafter ‘users’) of the Brazilian Unified Health System (SUS) and the possible implications for AMR. The aim is to map aspects that shape lay knowledge regarding antibiotics and AMR at the community level. We rely on Haenssgen et al.^8^ by defining ‘lay knowledge’ as the local, nonbiomedical notions of health formed within a specific cultural background. Our approach stresses how lived experiences are intertwined with one's cultural background. This intertwining serves as the context in which attitudes and practices related to antibiotic use and AMR understanding emerge.^9^, ^10^, ^11^ Patients' perspectives of past treatments should be acknowledged to enhance the success of actions to tackle AMR in health services.^12^ Analysis from this perspective is crucial to design and implement tailored actions of antimicrobial stewardship planned with community engagement^13^ and a social science approach.^14^ This exploratory analysis is part of a long‐term process which aims to support the implementation of the Brazilian National Action Plan (PAN‐BR)^15^ to tackle AMR. The article may also broaden the conceptual discussion regarding AMR by raising awareness about community members' perspectives.

To our knowledge, no previous qualitative study focusing on antibiotic use among users of Primary Care in Brazil has been conducted. This study was carried out at a Primary Care Unit (PCU) of the SUS in the area of Campo Limpo, a suburb of São Paulo. The PCU delivers free, public health services to the local population. It has 10 Family Health Strategy (FHS) teams, each comprising one physician (a general practitioner or an FH specialist), one nurse, one nurse technician and five community health workers. Community health workers are responsible for facilitating the users' access to health services and accompanying their health conditions. Each FHS team covers different territories within the surrounding region where the PCU offers health services.

## 2 METHODOLOGY

This study is part of a broader, qualitative project using the One Health approach to explore the perspectives of those involved in the demand and supply of antibiotics (*N* = 76). It examines aspects of antibiotic use at the community level among users; prescription practices among antibiotic prescribers, dispensers and other healthcare professionals (HP) in Primary Care; and the development of AMR policy in Brazil among policymakers, researchers and other stakeholders.^16^ The perspectives of the three groups of participants are integrated to provide a holistic view of the social dimensions for tackling AMR in the country. In this article, we focus on the perspectives of 19 users of the SUS, as they are generally underrepresented in biomedical or governance research. The number of participants was defined primarily by the saturation of responses related to the main domains of the study,^17^ which was discussed with the research team; also, the COVID‐19 pandemic in Brazil imposed restrictions on the face‐to‐face interactions between the interviewer and the participants. The project included observation of the local environment (general field notes by the first author) combined with in‐depth interviews. The aim was to explore closely the societal reality of the community covered by the healthcare service of the PCU.

### 2.1 Selection and invitation of participants

All interviews were conducted in Portuguese by the first author, who self‐identified as a cis‐male. He holds Master's and PhD degrees in Education and has skills in conducting journalistic and ethnographic interviews as well as experience in supervising qualitative research projects.

Purposeful sampling was used to select the participants, and users covered by the FHS implemented at the PCU. We presented the research scope and methodology to the PCU management team and community health workers of five different FHS teams. The community health workers proposed the names of users filling the criteria described below. The interviewer presented himself as a research assistant of a Brazilian University and invited participants by phone. Those who accepted were interviewed individually and privately at the PCU at a secure and discrete location. Interviews were conducted between August and October 2021. All hygiene rules adopted by the local Secretary of Health were followed to preserve the safety of both the interviewees and the interviewer. Seeking diversity in the participant sample, we sought users from different regions within the territory covered by the PCU, as well as a variety of gender and age. Additional inclusion criteria included (a) age over 18; (b) active registration at the PCU; (c) having attended the PCU at least once in the last 2 years. Due to the last criterion, most respondents were female, as women represent the majority of users within SUS.^18^

### 2.2 Data collection and analysis

This study consists primarily of individual in‐depth interviews. We developed a comprehensive interview guide for in‐depth interviews before starting data collection. It covered three broad domains: (1) how users understand their health conditions and how they deal with medication (including the features and use of antibiotics); (2) users' relationships with HPs at the PCU and (3) users' understanding of the risk of AMR, and how and by whom that information is communicated, as Appendix show. The interview guide was developed from both questions from previous qualitative studies and from an informational needs analysis of the project's broader international research team. It was not tested before the first interview, but questions were refined throughout the data collection process, as needed. Each interview lasted approximately 1 h. Three participants did not show up at the scheduled time for the interview, ceasing communication with the interviewer without reporting their reasons. There were no repeated interviews with users. The digitally recorded audio was transcribed verbatim in Portuguese by the interviewer. All transcriptions were reviewed by the first author and by the sixth author, who is the scientific coordinator of the project. Excerpts used in this article were translated into English by the first and sixth authors of this article; the latter was a native speaker. Quotes were coded according to thematic content analysis.^19^ The first author conducted the line‐by‐line analyses and original coding of themes derived from the data. The second and third authors collaborated in the creation and refinement of codes. Ongoing discussions on the data and its coding were carried out with the research group throughout the project, ensuring consistency throughout the different areas of the study. Interviews were then reviewed to understand the ways interviewees framed their practices and AMR to develop key themes. These themes were structured separately and then grouped into major clusters, as explored in the results and discussion sections that follow. Microsoft Word was used to highlight relevant excerpts of each theme and subtheme. All relevant excerpts were then extracted to Microsoft Excel, and identified by the corresponding codes. Participants were coded by number, gender and age (e.g., R14, female, 58). Table 1 illustrates the themes and subthemes from which the interview quotes here presented were selected and categorized.

**Table 1:** Themes and subthemes derived from the data

Health conditions and antibiotic use	Antibiotics	Understanding of the features of antibiotics Understanding of potential harms of antibiotic use Following medical orientations to take antibiotics
	Other medications	Understanding of the differences between antibiotics and other medications Understanding of the features of other medications Frequency of use of other medications
Relationship with HP	Demand for consultation	Painful symptoms Accident Periodic healthcare consultation Healthcare level of access
	Valuable characteristics of HP during consultation	Educational skill Professional experience Ability to listen
	Information exchange during consultation	Prescription orientations Medication features Potential harms of general medication use
Understanding of the risk of AMR	Information exchanged with HP	Length of treatment and dosage of antibiotic Potential harms of antibiotics Understanding of HP language
	Knowledge gathered through personal experiences	Previous bacterial infections Children's caregiving and administration of antibiotics Shared narratives within the household and the community
	Information gathered on media	Health campaigns TV or radio Social media, internet

Abbreviation: HP, healthcare professional.

*Source*: Main author.

## 3 RESULTS

Respondents self‐identified their race in the following way: two as White, three as Black and 14 as mixed‐race Brown. Their education level was diverse, with seven having attended elementary school (completed or not), nine reaching high school (completed or not) and three reaching university level (completed or not). Participants were aged 19–62, and most were women (*n* = 17).

Our findings show the important role that users' experiences play in three dimensions related to antibiotic use: (1) lay knowledge about medicines; (2) previous bacterial infections and (3) communication during the consultation.

### 3.1 Dimension 1: Lay knowledge about medicines

Lay knowledge encompasses the respondents' understanding of how antibiotics work in comparison to other drugs, and the experimentation they do with medication.

In response to our general question about what they know about antibiotics and medication, users affirm an awareness of classes of medication, such as antibiotics, analgesics and anti‐inflammatory drugs. Their explanations about differences and applications vary. Some affirm that antibiotics are a stronger type of anti‐inflammatory (‘antibiotics, I think [it] is a little bit stronger than anti‐inflammatory but with the same properties’, R14, female, 58), or that antibiotics function as anti‐inflammatories but are more intense (‘anti‐inflammatory works for a deeper inflammation, antibiotics I also believe that should be more or less the same thing’, R17, female, 35). Others view the difference between anti‐inflammatories and antibiotics as a dichotomy between topical and oral medication: ‘anti‐inflammatory works for what? For inflammation. Antibiotics work for a chronic inflammation which is not external, it is something that should be taken care of from the inside out’ (R10, male, 61).

Additionally, most respondents operate their own symbolic categories of ‘strong’ and ‘weak’ medication according to prior experiences with pain, illness and its symptoms. The faster a medication cures painful symptoms, the stronger they consider it. If they do not perceive any positive effect from a medication, they consider it as ‘weak’. Antibiotics are considered ‘strong’ by most respondents, not only because they experience rapid healing (R15), but also because they must follow specific rules regarding the length of treatment and dosage (R16).

R: Amoxicillin is very strong, amoxicillin, diclofenac, and Dorflex.

I: And why do you consider amoxicillin strong?

R: Because I myself, in my opinion, when I take it, when I have a problem with my body, I quickly get better.

(R15, female, 50)

R: I consider amoxicillin strong because my mother … says we can't use it for [just] anything, right? […] amoxicillin for example, you have to take it for an exact number of days, it is not the same as Doril [a brand of analgesic] or something that we take once and that's it, it has to be scheduled […] it's a rule, so as it has more restrictions, we already give it more importance, so I think it's stronger.

(R16, female, 19)

We asked respondents about their understanding of relevant terms related to AMR. Respondents were not uniform in their understanding of ‘resistance’. For instance, when R19 was first asked about ‘bacterial resistance’, she did not recognize the term. However, ‘antibiotic resistance’ was familiar to her. Her understanding of antibiotic ineffectiveness comes from a deduction made from a conversation within the household on the functioning of dipyrone (anti‐inflammatory) as compared to antibiotics.

I: And have you heard about bacterial resistance?

R: No, no.

I: Antibiotic resistance?

R: I think it's when you've taken the same medicine many times and then the medicine no longer has the effect it would […]

I: Do you remember from where you heard about this antibiotic resistance?

R: Once, at home, everyone talking as a family, then I have an aunt, who is crazy for dipyrone, right, then my cousin said to her, ‘Mom, stop taking dipyrone, soon it won't have effect on you’, then later I deduced that with all medicines it is the same thing.

(R19, female, 20)

Respondents assemble an array of lived experiences to deal with their health problems. As a result, they may not seek professional care if they feel they have resources at home that may treat their condition. Some respondents state they have used medication and chemical products to treat specific diseases based on perceived properties that, in their understanding, have healing potential. Those practices arise from their experiences in using medication and through shared narratives about healing within their network and serve as the basis for the rationale they present in choosing these nonconventional solutions. The first excerpt involves using vaginal cream for an inflamed inner ear, and in the second the respondent uses creolin (an environmental disinfectant) to combat gastritis:

R: I feel like I'm going to get the flu, my ear starts to itch, it gets inflamed, it turns red, […] Then, do you know what I put on? I take the swab, I get vaginal cream, I put it on the swab, I put it inside my ear, can you believe it?

I: But why the vaginal cream?

R: Because the vaginal cream is good, because if we have a wound, if we have an infection, we put it on, right? It eliminates the pain and everything from us. And in the ear it's the same thing, it's an internal place, a place where you can put the medicine, it inflames, we can't see it, it is the same thing when we have a problem in the vagina, we put the medicine, the ointment, to relieve it.

I: How did you discover this?

R: By myself. […] I didn't have to go to the doctor.

(R6, female, 62)

R: Gastritis, you've heard of gastritis, right? So, I know a perfect remedy, good for gastritis […] do you know what creolin is? … a friend of mine … told me that he had gastritis that was turning into ulcers […] then someone told him that he could take creolin […] well, it makes sense because we use creolin here in the big city more as a disinfectant, […] my father used [creolin] to kill infection in animals […] and soon the animals' wounds would heal, so I said ‘if it was good, if it would heal those wounds and also ulcers, gastritis is a wound, so if you take it, it will definitely heal’.

(R8, male, 61)

### 3.2 Dimension 2: Previous bacterial infections

Respondents' narratives about bacterial infections can be divided into situations of urinary tract infections (UTIs) and antibiotic treatment for other conditions.

Respondents' knowledge about bacterial diseases comes primarily from UTIs, which may reflect the predominance of female participants in our study. Patterns of antibiotic use are associated with narratives of UTIs that encompass the search for healthcare assistance in hospitals, gender positionality, ageing and difficulties during treatment. Moreover, respondents also highlight memories of frustration when the treatment is ineffective. R7 says she became frustrated because her grandmother was ill, and ‘they [HPs] couldn't find the right medicine to fight the bacteria she had. […] the three courses of medication didn't fight the bacteria’ (R7, female, 41). In a second case, R11 recounts the story of the mother of her mother's ex‐employer, who:

had bacteria in her urine […], and then the doctor couldn't treat it, she did several, and several, and several surgeries and [they] didn't cure it, she was in a wheelchair and that was not cured yet, then I heard that the urinary tract infection bacteria is very strong. […] people talk about the urinary tract infection bacteria.

(R11, female, 35)

In the following excerpt, R14 reveals her frustration with the prolonged need for care and notes that the HPs may lose track of the treatment history to date. R14's experience also reveals tensions that may arise between users and professionals, which leads to frustration:

R: My mother had a urinary tract infection for a long time, […] every Saturday I would go to the healthcare service with her, and the doctors would prescribe medication, or give medicine, it would be resolved for 2, 3 days and then come back, then one day I arrived at the hospital and said ‘look, young man, we need to know what is the bacteria that is making my mother like this, let's make a more complex test to be able to treat the bacteria, right’ […] I said ‘here, look at the prescriptions, she already took them, and I don't want to come to the doctor with my mother every week, so I want you to ask for an exam, a urine culture for her to be able to treat the bacteria that is leaving her with this pain’.

(R14, female, 58)

Previous antibiotic experiences provide a relevant set of narratives that may influence the way respondents use that medication in their present time. Despite rapid healing, discussed in Dimension 1, harms from antibiotics are also considered:

R: […] the memory I have is when you usually take antibiotics, depending on the antibiotic you are taking, the doctor even says ‘don't take them on an empty stomach’, the excessive use of antibiotics also harms the teeth, you know, of the child […] from what I have already experienced, being close to people that this happened to, children who were very sick in childhood, the color of their teeth began to change because the drug is very strong.

(R17, female, 35)

R3 mentions that her information about that topic came from her mother but was confirmed by her own experience in taking care of her daughter, who had bronchiolitis as a child.

I: And has a doctor ever told you about the consequences of using antibiotics or not?

R: No. Never. I always knew it causes some harm, right, in children. My daughter, she took a lot of antibiotics when she was young, because she had bronchiolitis, all that stuff, right? […] My mother always said that it ruined my teeth, weakened them, that sort of thing. But not because the doctor told me so.

I: It was from your mother's comments?

R: Yes.

(R3, female, 52)

In presenting experiences of female family members with UTIs, respondents also acknowledge their having witnessed treatment difficulties, harm associated with antibiotics and tensions with HPs.

### 3.3 Dimension 3: Communication during the consultation

Communication during consultation is characterized by a lack of shared knowledge and trust in the doctor‐user relationship.

As elements shown in Dimension 2 above suggest, respondents have their own knowledge about medication use, antibiotics and bacterial infections. However, they feel there is no opportunity to share their knowledge during the consultation. HPs communicate basic information when prescribing antibiotics, such as treatment length and dosage, but users report that HPs seldom provide guidance about possible harm from the medication, nor do they ask users what they already know about AMR. It creates a communication gap:

I: What about the consequences of eventually taking [antibiotics?]

R: No, never, he [the doctor] just told me that it was the deadline I had to use until the seventh day. And if necessary, to continue the treatment, it had to be guided by them. That's all.

I: So, information on bacterial resistance, antimicrobial resistance?

R: No, no, never, never spoke about and I never asked either, no.

(R4, female, 43)

One explanation provided for the low quality of information exchange between the users and the HPs is the short time allocated to the consultation (15 min). Another aspect hindering good communication is the shame some users feel when asking questions:

R: Because there are moments when the consultation is very fast, you know? And there are moments when the doctor also doesn't give us the opportunity to ask, then we ask something, then we feel ashamed to ask the next question.

(R3, female, 52)

Additionally, some respondents reveal their awareness of the high demand for health services and do not want to overstay their allotted time:

R: I know that sometimes the doctor, when he is not so thoughtful, […] when he does not have so much time to talk to the patient, it is not because he doesn't want to, it is because he has to respect the demand for scheduled appointments [at the PCU] because after you there can be someone with a more serious problem, you have to understand that.

(R17, female, 35)

Aspects of the doctor–patient relationship are evoked as relevant to determine whether there will be trust in the HPs' advice. Respondents' perception of the doctor's attitude towards them also plays a decisive role in following (or not) the doctor's advice (R17).

Also, being able to express feelings is important for the respondents, as they feel they ‘know their own bodies’ better than the HP (R10).

I: And do you always follow all the advice the doctors give?

R: So, […] when I feel it's true, yes […] because you know your own body, nobody better than you to know if you're okay or not […] if the doctor doesn't care about you, there are doctors who don't even look at your face, […] he doesn't know what is really going on with you.

(R10, male, 61)

R: Look, I follow [the doctor's orientations], but there are things I don't follow so strictly, it's not that I don't trust in what the doctor is saying, he studied for that […] but sometimes the doctor says some things that are not part of what you're feeling, […] sometimes you can come to a PCU like this one […] and suddenly the doctor is so rude, so gross, [she/he comes with] the prescription in hand and you'll say ‘I'm not going to take this [medication] that he prescribed because I don't know if it is really part of what I'm feeling’.

(R17, female, 35)

## 4 DISCUSSION

Our findings illustrate how users build their knowledge from their own experiences, which shape their understanding of antibiotic use, bacterial infections and AMR. We suggest that these experiences are interwoven with the information received from HPs on these topics, indicating that professional information about antibiotic use and its implications shared during the consultation is not the only source of users' ‘lay knowledge’. In line with other scholars,^8^, ^9^, ^10^, ^12^, ^20^, ^21^, ^22^, ^23^ our analysis shows that respondents rely on a set of experiences and values embedded in their cultural settings that shape both antibiotic use and knowledge about AMR, and users develop an important sense of autonomy about medication and their own bodies in the intertwining context of health experiences and information gathered within their community network.

Respondents integrate their antibiotic experiences into their knowledge about other drugs, such as analgesics and anti‐inflammatories, as other studies have shown.^3^, ^24^ They claim that antibiotics are ‘strong’, setting them apart from other drugs, based on the duration, dosage and frequency of treatment.^23^ Respondents show they know antibiotics have specific features, as they believe that antibiotics work to cure ‘chronic internal inflammation’ in the body and aid in rapid recovery. These results parallel those of other studies in that users perceive that antibiotics are a special type of drug.^23^, ^25^, ^26^

Despite viewing antibiotics as ‘strong’, some respondents forgo their use, choosing instead alternative practices completely outside standard medical care (e.g., off‐label use of vaginal cream, creolin). If these alternative practices seem to work, these experiences reinforce the users' sense of autonomy in dealing with their own health and reliance on lived experiences and shared narratives of healing. These practices indicate a mixed knowledge frame between lay knowledge and biomedical information and suggest cultural entanglements in which antibiotics are intertwined for the respondents.^9^ Our study shows that some of these entanglements come from comparisons with other drugs, previous use of antibiotics and experiences of illness that are shared within households and the community‐level network. Because of these practices, respondents' current ideas about the ‘appropriate’ use of medication, like antibiotics, can be resignified through the negotiation between lay knowledge and professional information.^11^, ^20^, ^23^

The issue of gender is important to our article in three ways: first, studies investigating gender, antibiotic use and AMR as articulated themes are scarce.^10^, ^11^, ^27^ Second, antibiotics are prescribed in Primary Care more often to women,^11^ who are portrayed as having acquired better knowledge about antibiotic use and AMR.^27^ Third, the role of women as family caretakers appeared to be relevant in our study. This central role, combined with the high incidence of bacterial infections (particularly to treat UTIs^28^) among women, suggests that a focus on female users provides a relevant dimension to better map the sociocultural context that shapes antibiotic use.^10^, ^11^

Lived experiences with bacterial infections which are not properly treated can lead to tensions in the relationship with HPs. As repeated diagnostic procedures and the use of inadequate antibiotics do not cure a given health problem, there is a feeling of failure and confusion among users (cf. Boiko et al.^29^).

There may be a ‘grey area’^28^ in the communication between HPs and users, as neither seems to address differences between medications or their potential harm.^30^ This ‘grey area’ may be a result of structural constraints, such as the limited time for sharing information between HPs and users^31^ and the high demand for consultations at PCU, but it is also related to the feeling of trust users have regarding the quality of their relationship with HP during the consultation. The users' assessments will influence how their consideration of and degree of compliance with doctors' instructions. In fact, users affirm they often let their experiences take precedence over professional information, because they trust their knowledge about medication, antibiotic use and bacterial infections, and their personal narratives have not been heard by the FHS team. In contrast to other studies,^32^ our findings show that users expect the HP to listen to their experiences during consultation rather than providing pieces of information which are disconnected from their day‐by‐day life.

Our results align with those of Haenssgen et al.,^8^ whose approach to the use of antibiotics and AMR explores the lay knowledge and the ‘tales of treatment’ that are shared at the community level. The medical consultation is one moment, among others, in which information on medication use can be exchanged, sometimes competing with narratives, experiences and previous ‘tales of treatment’ of the users. Thus, even if doctor–patient communication is relevant, the information shared is not necessarily the most important for the respondents: information is assembled with other health experiences and can be relativized, ignored or contested.

The respondents' expectation is that the consultation is a moment of proximity with HPs, as other studies also pointed out.^23^, ^33^ Once the respondents effectively build a repertoire of experiences about antibiotics use and health issues, their expectation is that HPs will listen to and respond to these experiences.^12^, ^34^ Compliance with medical orientation, as the respondents' state, is linked to the trust they have in the doctor, paralleling recent research.^30^ Thus, active listening to users' experiences can help HPs identify the specific contexts in which they make decisions on following or not medical instructions.^10^

Trust is renegotiated at every consultation and requires that the HPs acknowledge the users' assessments of ‘their own bodies’. Also, even if the evaluation of medical care is positive, the idea that everyone ‘knows their own body’ prevails among users. ‘Knowing one's own body’, in this sense, is something different from the apprehension of biomedical knowledge (e.g., diagnosis and appropriate treatment); it is about the possibility of expressing what one feels through the body, and how these feelings refer to previous experiences of medication use and illness. For the respondents, shared narratives in the community and lived experiences constitute what they call ‘knowing one's own body’. In this sense, they merge experiences to build a knowledge frame related to medication, antibiotic use and bacterial infections, through which they come to understand AMR. Eventually, they also apply this knowledge frame to negotiate compliance with medical orientations and to ascribe their own meanings to the potential harms of antibiotic use.

One strength of our study is fostering the appreciation of qualitative aspects of antibiotic use in Brazil by continuously exploring three complementary perspectives: users, antibiotic prescribers and dispensers and policy stakeholders, to comprehend the views of all actors involved in antibiotic use in Brazil from a holistic perspective. It also adds information in qualitative research of themes concerning AMR in the country, where there are scarce studies of this type. Regarding the Brazilian NAP,^15^ our findings support the view that acknowledging the role of community members is fundamental to the success of its implementation, as opposed to taking a purely top‐down approach to policy development. We further suggest that the resulting interventions (e.g., local health promotion campaigns and tailored, specific educational training for HP of the FHS team) would be more impactful within the community if they incorporate the values of the communities they represent. Finally, our findings may not be generalized to the national level or abroad, although parallels can be found in other contexts.

## 5 CONCLUSION

Our study underlines how lived experiences are intertwined with professional information about antibiotics for users in Brazil. We adopt an approach different from most of traditional KAP research, as we investigate narratives of how these attitudes and behaviours are constructed instead of portraying them as single actions, disconnected from the users' contexts. Their sets of experience play an important role in healthcare, sometimes determining noncompliance of medical orientations and allowing unexpected uses of medication. The experiences of bacterial infections and their treatment, shared within the household and the community, are an important source of knowledge about antibiotic use and AMR among users. Users demand from their HPs both trust and willingness to listen to their health narratives and experiences. Still, users recognize the structural constraints that limit their communication with HPs, like the time allotted for each consultation and the burden of the public health system, both of which affect the quality of the information exchange.

Recognition that users have autonomy in deciding whether to follow medical advice or not and acknowledgement that this autonomy is based on users' sets of experiences may contribute to HPs' overall comprehension of users' attitudes and practices regarding their own health and bodies. Ongoing learning of users' experiences and understanding of antibiotic use is a shared responsibility among all HPs and is not restricted to information‐sharing in a single consultation. Users ascribe symbolic meanings to antibiotics and learn about AMR through lived experiences of bacterial infections, creating a lay knowledge frame, even if this knowledge is not scientifically correct. By considering lay knowledge as part of the assessment of a user's health condition, rather than dismissing it as erroneous and therefore unworthy of attention, HPs may enhance compliance of users in efforts to tackle AMR.

## AUTHOR CONTRIBUTIONS

Luiz F. Zago has conducted all interviews and has them transcribed. Luiz F. Zago, Juliana S. Correa, Roberto R. da Silva‐Brandão and Sandi Michele de Oliveira created the interview guide, interpreted and analysed data. Gloria C. Corboda Currea and Sandi Michele de Oliveira have created the research project and methodological design. Maria Clara Padoveze and Lislaine A. Fracolli have contributed to the research programme and worked as local academic liaison for the development of fieldwork. Roberto R. da Silva‐Brandão and Juliana S. Correa have contributed by summarizing the main contributions of the study. Luiz F. Zago and Sandi Michele de Oliveira have written the first draft of the manuscript. All authors drafted the interview guides at different stages of the research. All authors commented on subsequent versions of the manuscript. All authors have read and approved the final manuscript.

## CONFLICT OF INTEREST

The authors declare no conflict of interest.

## ETHICS STATEMENT

The research project was submitted to the Research Ethics Committee of the School of Nursing, University of São Paulo and to the Brazilian National Research Ethics Committee (CONEP) under the number CAAE 42442921.7.0000.5392. All participants were instructed about the objectives of the research project and were informed about the findings, following recommendations of the local research ethical committee. Participants are anonymous and the interviews were conducted upon their consent, registered on the Free and Informed Consent Form.

## Data Availability

The data that support the findings of this study are available on request from the corresponding author. The data are not publicly available due to privacy or ethical restrictions.

## LIST OF DOMAINS & QUESTIONS

### 1

This document presents the domains to be covered in the interviews associated with each of the outcomes laid out in the project proposal.

This is not the interview guide itself.

**WP 1: The individual, the household and the community**

**Preliminary questioning—warm‐up, general information**

- Household and the community
  ■ Number of years in the community
  ■ Number of years as patient at the health center in Campo Limpo
  ■ Number of people in the household
  ■ Number of pets in the household (what are they?)—Additional questions on where the pet sleep, if the pet wander outdoors, what the pet eat, etc.
  ■ Meaning of the relationship between humans and animals (psychological health and wellbeing by providing companionship, emotional and social support, a sense of safety and security, entertainent, happiness and relaxation)
- Well‐being
  ■ Self‐assessment of wellbeing of self and other members of the household
  ■ Knowledge (and sources of knowledge) regarding healthcare and assumptions about what makes for good health
  ■ How important good health is in the household (and what they do about it)
  ■ When you (or your pet) are not feeling well, how do you decide when to go to the doctor/vet?
- Past experiences(s) with infections
  ■ Can you remember the last time you had an infection?
  ■ What type was it (urinary, respiratory, something else?)
  ■ Was it severe?
  ■ Were you prescribed an antibiotic?
  ■ Did you have to go to the hospital?
  ■ To your knowledge, have you been infected with COVID‐19? (How severe? Medication? Hospitalization?)

**Identification of the perception that patients and animal owners have regarding AMR**, its relevance to their lives and the information conveyed to them by the community (health providers, governmental agencies).

- Health information for the public
  ■ Where do you get information on health issues? (e.g., directly from doctors/nurses, health center posters or brochures, internet, parents/children, friends, TV, digital panels on the street, pharmacies, veterinarians, pet shops, etc.)
  ■ Which are your preferred sources for good information? Which do you trust most?
- AMR
  ■ Have you heard about antibiotic resistance or antimicrobial resistance? **(if yes, continue with these questions and then move to Antibiotics; if not, go straight to questions on antibiotics)**
  ■ What have you heard?
  ■ What do you understand the term ‘AMR’ to refer to?
  ■ Have you heard the term ‘antibiotic footprint’?
  ■ Have you heard that AMR can be transmitted between household members (including pets)? Do you remember where and from whom you heard it?
  ■ Do you remember any campaigns about AMR?
  ■ Did it have any impact on your knowledge or opinion about AMR?
- Antibiotics (in the interview guide, this section will precede the questions on AMR)
  ■ What do you know about the way antibiotics work?
  ■ Do you know when they should and should not be prescribed?
  ■ (if not already asked) Are you aware that overuse of anitibiotics can lead to AMR, which can be transmitted to others in your household and beyond?
  ■ Have you heard the term ‘critically important antibiotics’?
  ■ Ability to recognize the names of some common antibiotics that the doctor might mention
  **(if during the conversation they mention remembering having heard about AMR, circle back and ask what they know)**
- Awareness of efforts to improve health care in Brazil (by viewing the connections between human and animal health in their shared environments)
  ■ Local & Regional levels (to build up to the national)
    ■ Local efforts to improve healthcare (e.g., organized by health authorities, local associations)? Have you participated?
    ■ What do you see as the most important issues involving the health of the local community? (can expand to issues that can impact health generally, such as waste disposal, pollution, etc.)
    ■ At the municipal level have you/your family/friends ever gotten involved with the Conselho Participativo in Campo Limpo? (if so, what were the principal issues of interest?)
    ■ Are you aware of any (or any other) efforts to improve the health of the community by examining the relationship between people, animals and the physical environment?
  ■ National level
    ■ Awareness of the existence of the NAP (if yes, through what means?)—mention attempts to make a better NAP
    ■ ‘National plans are designed to change the way each of us behaves and the way our behavior is monitored. If you could speak with the policymakers directly about the health issues that affect you and your household, what would you like them to know about?’ (this has to be built up slowly; not every patient will likely be able to discuss this at length)

**Identification of the ‘One Health’ dimensions** involved in the inappropriate use of antibiotics at the individual, household and community levels.

- Antibiotic use/responsible use of antibiotics **(the starting point is the patient's personal experience)**
  ■ Do you remember the last time you were prescribed an antibiotic?
  ■ What were you sick with, and how long did you take the medicine?
  ■ Do you remember what antibiotic it was/do you have the package it came in?
  ■ Was the antibiotic prescribed at your first consultation for this illness, or were you asked to come in for a second consultation before the antibiotic was prescribed?
  ■ Have you ever been given a prescription but told not to fill it until several days had passed?
  ■ Do you remember what antibiotic it was/do you have the package it came in?
  ■ Knowledge
    ■ What did the doctor say to you when prescribing the antibiotic?
    ■ Did he/she give you any written information to take home?
    ■ Did anyone else at the health center give you supplementary information on using antibiotics?
    ■ Did the nurse/pharmacist tell you about using this medicine?
    ■ Were you told about how to store and later dispose of antibiotics?
    ■ **(if have pets, the same questions relative to a vet/pet shop)**
  ■ Compliance
    ■ Did you take the antibiotics for the entire length of time the doctor prescribed?
    ■ Were there any antibiotics left over? If yes, what did you do with them?
    ■ **(same questions with regard the antibiotics with pets)**
  ■ Self‐medication/alternatives to antibiotics
    ■ Does access play a role (distance and affordability)
    ■ Do you try to diagnose yourself or other household members (including pets)?
    ■ Other methods of obtaining antibiotics: Internet pharmacies, asking a doctor ‘friend’ to issue a prescription, saving up from a previous infection, pet stores (for antibiotics for humans)
    ■ Sharing antibiotics with household member(s)
    ■ Have you ever gotten an antibiotic without a prescription? How easy was it? Was it from a pharmacy or through some other means?
    ■ Do you ever or have you ever sought help from alternative medicine (what? When? For what ailment? etc.)—can ask about óleos essenciais [essential oils], probióticos, prebióticos, others?

**Users'relationships with healthcare professionals at the PCU**

- Doctor–patient encounter (consultation)
  ■ How prescribers handle the treatment phase of the consultation (e.g., through mandate, discussion/negotiation)
  ■ Do you prefer written or/and oral information support tools to receive the message from their doctor/nurse/pharmacist?
  ■ Have you talked with your doctor/the vet about the benefits and harms of antibiotics?
  ■ Have you talked with your doctor/the vet about AMR?
  ■ Strategies that patients/pet owners use to request or reject the use of antibiotics.
  ■ Do you feel your doctor/vet is empathetic? **(if yes)**
    ■ How is this manifest (or not)?
    ■ Are you more likely to follow the doctor's/vet's recommendations if they are empathetic?
